# Tension across adherens junctions: when less is more

**DOI:** 10.18632/oncotarget.5448

**Published:** 2015-09-01

**Authors:** Kevin Kruse, Yulia A. Komarova

**Affiliations:** Department of Pharmacology and The Center for Lung and Vascular Biology, University of Illinois at Chicago, College of Medicine, Chicago, IL, USA

**Keywords:** tension, adhesion, VE-cadherin, RhoGTPase, junction

**Figure 1:**
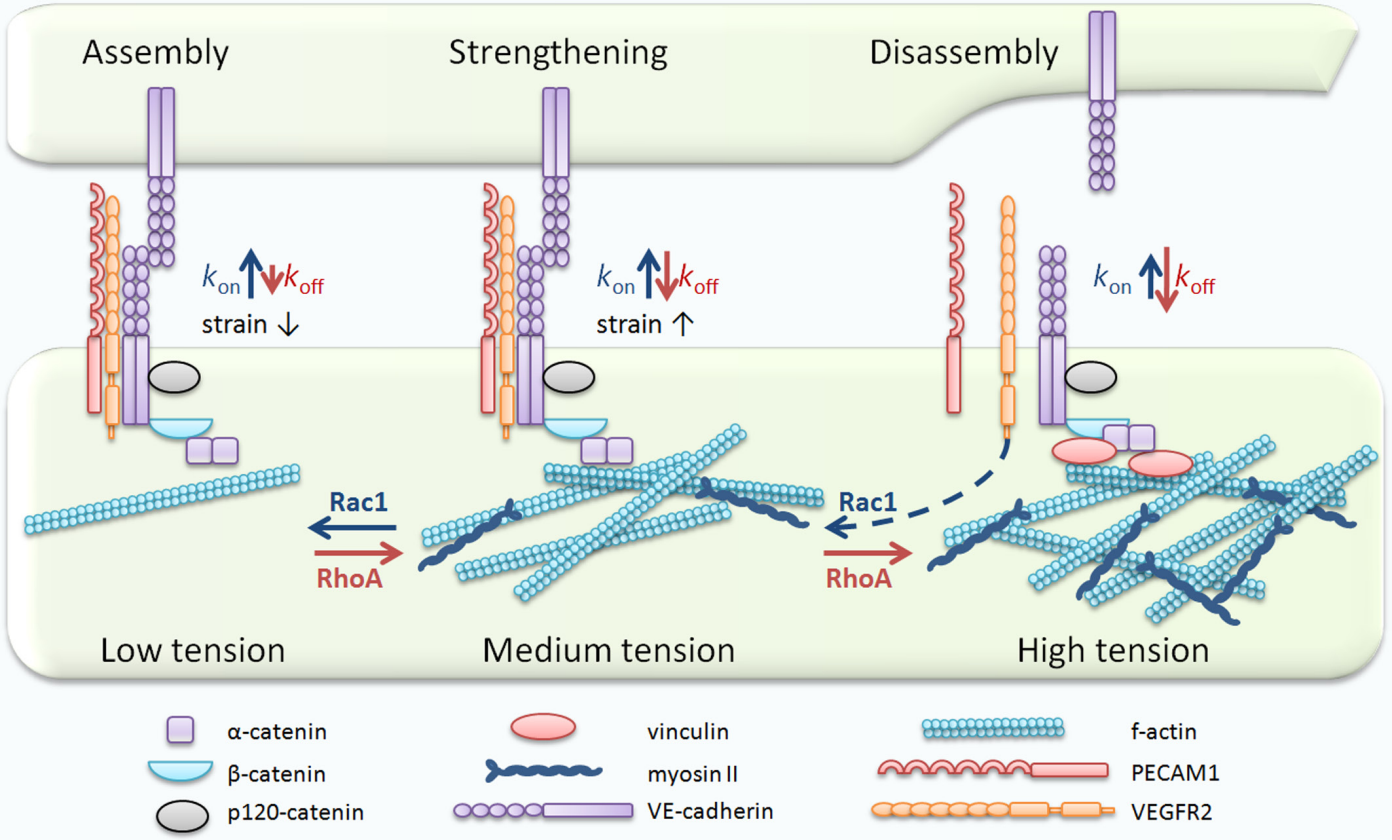
Relationship between tension and stability of VE-cadherin adhesions VE-cadherin *trans*-dimerization promotes assembly of AJs whereas attachment of the VE-cadherin–catenin complex to the actin cytoskeleton increases the strength of AJs in a tension-dependent manner. Low tension promotes formation of stable bonds between the VE-cadherin–catenin complex and actin filaments whereas high tension disrupts VE-cadherin *trans*-interactions, inducing VE-cadherin dissociation from AJs. Tension also promotes conformational changes in α-catenin, allowing for vinculin binding. Recruitment of vinculin to AJs induces re-distribution of tension while disruption of the mechanosensory complex with VEGFRs results in activation of Rac1, inducing reassembly of VE-cadherin adhesion.

The selective permeability of the endothelial monolayer is controlled by adherens junctions (AJs), which connect endothelial cells through adhesion of Vascular Endothelial (VE)-cadherin ectodomains (EC) in a strand-swap configuration, an anchorage of the conserved tryptophans Trp2 and Trp4 to a hydrophobic pocket of the opposing EC1 domain. This adhesion is a dynamic event characterized by the association (*k*_on_) and dissociation (*k*_off_) rates of the extracellular moiety of VE-cadherin [1] (Figure 1). We have recently demonstrated that mutation of Trp2 and Trp4 significantly increases the VE-cadherin dissociation rate from AJs [1], suggesting that *trans*-dimerization “traps” VE-cadherin molecules at AJs. Hence, assembly of VE-cadherin adhesive bonds induces recruitment of VE-cadherin molecules to AJs by a *diffusion trap* mechanism to restrict permeability of the endothelial barrier to macromolecules.

The strength of VE-cadherin adhesive bonds, defined as the ability to sustain mechanical tension, requires attachment of the VE-cadherin adhesion complex to the actin cytoskeleton in a tension dependent manner [2]. In elegant studies by Buckley and colleagues, the interaction between the cadherin-β/α-catenin complex and actin filaments was explained by a *two state catch bond* model in which the dissociation rate of F-actin from the cadherin complex decreases exponentially with respect to applied tension [2]. Using an optical trap-based assay, the authors showed that the cadherin-catenin complex forms stable bonds with F-actin filaments <10 pN whereas higher forces promote dissociation of F-actin from the complex [2]. These *in vitro* data and our most recent findings [1] suggest that assembly and strength of VE-cadherin adhesion is finely regulated by myosin-II-generated intracellular tension. This tension, depending on its distribution, nature, and magnitude, can favor VE-cadherin adhesion assembly or disassembly *via* modulation of the VE-cadherin dissociation rate (Figure 1).

Proteins of the adhesion complex not only provide a mechanism for cell-cell adhesion but also serve as sensors and transducers of mechanical tension. Sensors such as VE-cadherin and platelet endothelial cell adhesion molecule (PECAM-1) “perceive” intracellular forces [3,4], whereas transducers such as α-catenin and vinculin transmit force [5]. At mature junctions, α-catenin undergoes rapid and reversible switching between tension states, suggesting the transient nature of traction forces [5]. This might be a result of burst activation of intracellular signaling molecules such as the RhoGTPase Rac1, which we have shown to promote assembly of *trans-*dimers by altering RhoA and myosin-II activity and reducing tension across AJs [1]. Our model proposes that the relative activation levels of RhoA and Rac1 are essential drivers of assembly, stabilization, and remodeling of VE-cadherin adhesive bonds at mature AJs (Figure 1). RhoA promotes activation of myosin-II in a ROCK-dependent manner, which in turn generates intracellular forces needed for formation of stable bonds between actin filaments and the VE-cadherin complex. Rac1 counteracts these forces to promote VE-cadherin *trans*-dimerization by reducing the VE-cadherin dissociation rate and sequentially increasing VE-cadherin density at AJs. An increase in traction forces across VE-cadherin adhesions triggers prolonged conformational changes in α-catenin leading to recruitment of vinculin to AJs [5, 6]. This sequence of events might play a critical role in redistribution of localized forces. Vinculin can promote actin remodeling *via* Mena/VASP and Arp2/3-dependent pathways and tune assembly of AJs independently or in concert with Rac1 [6]. Hence, in our model, Rac1 promotes assembly of VE-cadherin adhesions whereas RhoA increases adhesion strength. This ‘*tug of war*’ between RhoA and Rac1 signaling at AJs is an important mechanism regulating the plasticity of VE-cadherin adhesions and adaptation of endothelial cells to environmental cues.

The ability of cells to sense their environment is a critical factor in a variety of vascular processes such as adaptation of endothelial monolayers to fluid shear stress (FSS). VE-cadherin forms a junctional mechanosensory complex with PECAM-1 and vascular endothelial growth factor receptors (VEGFRs) [3, 4]. FSS induces ligand-independent activation of VEGFR signaling [4], resulting in decreased tension across VE-cadherin adhesion [3], possibly through activation of Rac1. FSS also promotes association between PECAM1 and vimentin [3], an intermediate filament protein. The force-activated reorganization of intermediate filaments leads to redistribution of mechanical forces and prevents destabilization of VE-cadherin adhesions. This positive feedback mechanism plays a critical role in the adaptation of endothelial cells to environmental cues to help maintain barrier function and tissue homeostasis.
